# The absence of BBSome function decreases synaptogenesis and causes ectopic synapse formation in the retina

**DOI:** 10.1038/s41598-020-65233-4

**Published:** 2020-05-20

**Authors:** Ying Hsu, Janelle E. Garrison, Seongjin Seo, Val C. Sheffield

**Affiliations:** 1 Department of Pediatrics, Division of Medical Genetics and Genomics, University of Iowa Carver College of Medicine, 4181 MERF, 375 Newton Road, 52242 Iowa City, Iowa United States; 2 Molecular Medicine Graduate Program, University of Iowa, Iowa City, United States; 3 Department of Ophthalmology and Visual Sciences, University of Iowa, Iowa City, Iowa 52242 United States

**Keywords:** Neurodevelopmental disorders, Genetics of the nervous system, Synaptic transmission, Retinal diseases, Genetics research

## Abstract

Photoreceptors possess ribbon synapses distinct from the conventional synapses in the brain. Little is known about the function of the BBSome, a complex integral in ciliary and intracellular trafficking, in ribbon synaptic formation. We performed immunohistochemistry using retinas from Bardet-Biedl Syndrome (BBS) mouse models and found that BBS mutant animals have significantly fewer ribbon synapses in the outer plexiform layer and increased ectopic synapses in the outer nuclear layer compared to controls. Many ectopic synapses in BBS mutant retinas are associated with horizontal cell axonal processes that aberrantly intrude into the outer nuclear layer. To determine whether this horizontal cell phenotype is a consequence of retinal degeneration, we examined this phenotype in mice with photoreceptor-specific inactivation of the BBSome induced by *Cre* recombinase driven by the rhodopsin promoter. At three months of age, despite retinal degeneration, *Bbs8*^*floxed/floxed*^*; Rho-Cre*^+^ mice lack the aberrant intrusion of horizontal cell processes. At 6 months, some horizontal cell processes intrude into the outer nuclear layer in *Bbs8*^*floxed/floxed*^*; Rho-Cre*^+^ mice, but the phenotype does not recapitulate the phenotypic severity observed in young congenital BBS mutant mice. Therefore, the lack of BBSome function negatively impacts retinal synaptogenesis, and causes horizontal cell defects in a potentially cell-autonomous fashion.

## Introduction

The BBSome is a protein complex consisting of BBS1, 2, 4, 5, 6, 7, 8, 9, and 18^1–9^, and specializes in ciliary transport. Functioning as an adaptor to the intraflagellar transport (IFT) complex^10^, the BBSome expands the cargo range of the IFT complex in ciliary transport^11^. In cilia of *Chlamydomonas reinhardtii*, single-particle imaging reveals that a BBSome cargo phospholipase D comigrates with the BBSome on IFT trains^11^, and the loss of BBSome function causes accumulation of phospholipase D in cilia^10^. The disruption of BBSome function in mammalian cilia likewise causes accumulation of cargoes in cilia including Smoothened^12^, Patched 1^12^, and Dopamine receptor^13^, all requiring the BBSome for ciliary export. In addition to the retrograde transport of cargoes^10^, the BBSome also enables their passage through the transition zone^14^.

In addition to its roles in ciliary transport, the BBSome also plays a role in non-ciliary intracellular transport. In many cases, the BBSome regulates protein localization to the plasma membrane. For example, the BBSome regulates the trafficking of the long form of the leptin receptor to the neuronal plasma membrane, influencing food intake and energy expenditure^15^. In addition, the BBSome physically interacts with the insulin receptor and regulates its trafficking to the cellular plasma membrane^16^. Besides from modulating trafficking to the plasma membrane, in a non-ciliated cell population in zebrafish, the inactivation of BBS genes disrupts the retraction of melanosomes, a dynein-dependent process regulating zebrafish pigmentation^17^. These reports demonstrate that the BBSome has cellular functions outside cilia. Additional novel functions of the BBSome have been identified. Recently, the BBSome was implicated in axonal targeting in neurons. Loss of one of the BBSome components, BBS8, adversely affects axonal targeting in the olfactory bulb^18^. Furthermore, the inactivation of BBSome function reduces dendritic lengths and the number of dendritic spines in neurons in the brain, implicating the BBSome in synaptic connections^19^. The role of the BBSome in synaptogenesis and in modulating neuron-neuron contact is a growing area of research that merits further exploration.

Photoreceptors are neurons in the retina that have evolved enlarged ciliary structures, the outer segments, which specialize in light detection by displaying an enormous amount of opsin protein on their neatly stacked membranous discs. The formation of the photoreceptor outer segments requires ciliary genes including those genes that encode BBSome components^20,21^. Loss of BBSome function in *Lztfl1 (Bbs17)* mutant mice causes outer segment malformation, as well as leads to the mislocalization of more than a hundred proteins into the photoreceptor outer segment^22^. Therefore, the BBSome is not merely required for ciliary trafficking in photoreceptors but is also indispensable for the proper structural formation, or morphogenesis, of the outer segments. In human patients and mouse models with mutated components of the BBSome, loss of BBSome function causes retinal degeneration, leading to blindness^13,20,21,23^.

In addition to having specialized cilia to support phototransduction, photoreceptors form synaptic structures called ribbon synapses, which are distinct from synapses in the brain. Due to their extraordinary requirement for transducing signals with speed and graded precision, ribbon synapses are uniquely designed for a large number of release-ready vesicles to dock at the active zone^24,25^. This efficient signal transduction between photoreceptors, bipolar and horizontal cells facilitates rapid and sensitive perception of visual stimuli. Anchored to the presynaptic membrane by the protein Bassoon (BSN)^26^, ribbon synapses contain ribbons, unique cytomatrix structures that provide substrate for the docking and tethering of a large number of vesicles proximal to the synaptic active zone. One of the major protein constituents of the synaptic ribbon is RIBEYE, a protein that contains an A domain that is specific to ribbon synapses and capable of forming detergent-insoluble aggregates, and a B domain that is identical to the transcriptional repressor CtBP2^24^. RIBEYE and CtBP2 are transcribed from the same gene, with CtBP2 showing wide expression in many tissues and RIBEYE expression restricted to a few tissues. The unique ability of RIBEYE to form ribbons is conferred by its aggregate-forming A domain, an evolutionary innovation in vertebrates^24^. The deletion of RIBEYE abolished all synaptic ribbons in mice and reduces the quantity of docked vesicles^27^. The deletion of *BSN*, on the other hand, causes free-floating ribbons^26^. It is not clear whether loss of BBSome function impacts the ability of the photoreceptors to elaborate ribbon synapses. In the study reported here, we investigate the formation of ribbon synapses among photoreceptors, bipolar and horizontal cells in *Bbs2*^−/−^, *Bbs4*^−/−^, *Bbs7*^−/−^, *Bbs8*^−/−^ and *Bbs1*^*M390R/M390R*^ mice to elucidate the role of the BBSome in synaptogenesis. Our results indicate that absence of BBSome function negatively impacts photoreceptor synaptogenesis and causes aberrant positioning of synaptic contacts between neurons.

## Methods

### Animal models and ethics statement

This study was performed in strict accordance with the recommendations in the Guide for the Care and Use of Laboratory Animals of the National Institutes of Health. All animals were handled according to approved Institutional Animal Care and Use Committee (IACUC) protocol #8072147 of the University of Iowa. Animals were housed according to IACUC recommendations. Both male and female mice were used in this study, and no sex differences were observed with regards to the phenotypes reported in this study. Methods of euthanasia used were carbon dioxide asphyxiation followed by cervical dislocation, or anesthesia induced by ketamine/xylazine followed by transcardial perfusion of 10% formalin. Humane endpoints were strictly observed, and every effort was made to minimize suffering. *Bbs2*, *Bbs4, Bbs7, Bbs8* knockout, and *Bbs1*^*M390R/M390R*^ knockin mouse models used in this study were published previously^13,20,23,28,29^.

Mice carrying the *Bbs8* floxed alleles, as well as *Cre* recombinase driven by the rhodopsin promoter (*Rho-Cre*) were generated from breeding mice carrying *Bbs8* floxed alleles^20^ and the *Rho-Cre* mouse line^30^. The genotyping for the presence or absence of the *Rho-Cre* was performed using GoTaq (Promega, Madison, WI) following the manufacturer’s recommendations. Primers used for genotyping *Rho-Cre* were described elsewhere^30^ and are listed as follows: F-Rho-Cre, TCAGTGCCTGGAGTTGCGCTGTGG; R-Rho-Cre, CTTAAAGGCCAGGGCCTGCTTGGC.

### Immunohistochemistry

Mice were anesthetized by intraperitoneal injection of a ketamine and xylazine mixture as previously described and transcardiac perfusion was performed using 10% formalin (approximately 4% formaldehyde) at 1.0 mL/min for a total volume of 1.25 mL/g body weight^20^. Eyes were enucleated, and a small puncture was created through the lens using a 26-G syringe. Eyes were then embedded in Tissue-Tek O.C.T. compound (VWR, Batavia, IL) and frozen in a 2-methybutane bath chilled with liquid nitrogen. Eyes were sectioned using a Cryostat microtome at thickness of 10 microns and stored at −80 °C for further use.

For immunohistochemistry, sections were permeabilized with 0.3% Triton X-100 in PBS for 10 minutes at room temperature, blocked in a blocking buffer containing 5% BSA, 5% Normal Goat Serum, and 0.05% Triton X-100 in PBS for one hour at room temperature, and incubated with primary antibodies at 4 °C overnight. The next day, slides were washed three times with PBS followed by incubation with secondary antibodies or streptavidin-Alexa Fluor 568 conjugate at room temperature for 1 hour. After another round of washing, slides were mounted with VectaShield anti-fade mounting medium with DAPI (Vector Laboratories, Burlingame, CA). Images were taken using a fluorescence microscope. Minimal image processing including contrast enhancement was performed using ImageJ.

Antibodies used for immunohistochemistry were as follows: anti-CtBP2/RIBEYE antibody (BD Transduction Laboratories #612044; 1:200 dilution); biotinylated-peanut agglutinin (biotinylated-PNA) (Vector Laboratories #B-1075; 1:500 dilution); anti-PKCα antibody (Sigma Aldrich #P4334; 1:1000 dilution); anti-calbindin antibody (Proteintech #14479-1-AP, 1:400 dilution); anti-neurofilament antibody 2H3 concentrate (Developmental Studies Hybridoma Bank, University of Iowa, 1:200 dilution); anti-syntaxin-3 antibody (Proteintech #15556-1-AP, 1:1000 dilution); anti-rhodopsin antibody 1D4 (Santa Cruz #sc-57432, 1:500 dilution).

### Quantification of retinal histology

Ten-micron thick retinal sections prepared from animals perfused with fixative as described above were processed for H and E histology and visualized under a light microscope. Images were acquired at 40X magnification. Thicknesses of retinal layers were quantified by an observer masked to genotypes using ImageJ. For each animal, two different images were analyzed, and the values were averaged for that animal. Thicknesses of retinal layers of wild type or heterozygous control were compared to those of knockout littermates using two-tailed *t* test with the assumption of equal variance. For performing statistical comparisons between multiple groups, one-way ANOVA with post-hoc Tukey’s test was performed. Error bars in graphs as well as range values denoted in descriptions represent the average ± standard error of the mean.

### Quantification of ribbon synapses

For the quantification of ribbon synapses in the outer plexiform layer, immunohistochemical experiments were performed in pairs containing one control and its knockout littermate. Images were acquired at 100X plus an additional 1.6X magnification and RIBEYE-positive, horse-shoe shaped synapses in the outer plexiform layer were quantified by an independent observer masked to genotypes. At least 4 different images were analyzed per animal to determine the synaptic density of that animal. Following quantification, the density of ribbon synapses per 100 μm in knockout animals is compared to that of their control littermates using two-tailed *t* test for paired data analysis in Microsoft Excel. Error bars in graphs as well as range values denoted in text descriptions represent the average value ± standard error of the mean.

For the quantification of ectopic synapses in the outer nuclear layer, the total number of RIBEYE-positive puncta in the outer nuclear layer was counted in three different images acquired at 40X with an additional 1.6X magnification per animal. The number of ectopic synapses per 100 μm width of the retina for that animal was derived. Then, the density of ectopic synapses in control and mutant animals were compared using two-tailed *t* test for paired data analysis in Microsoft Excel. Error bars in graphs as well as range values denoted in descriptions represent the average value ± standard error of the mean.

### Quantification of photoreceptor nuclei

For the quantification of photoreceptor nuclei, DAPI-positive nuclei located in the outer nuclear layer were counted by an observer masked to genotypes using 40X images of the retina for each animal. The number of photoreceptor nuclei in 100 μm width was determined and used for the normalization of ribbon synaptic number.

## Results

### *Bbs2*^−/−^ mice have a reduced density of ribbon synapses in the outer plexiform layer

**Figure 1:**
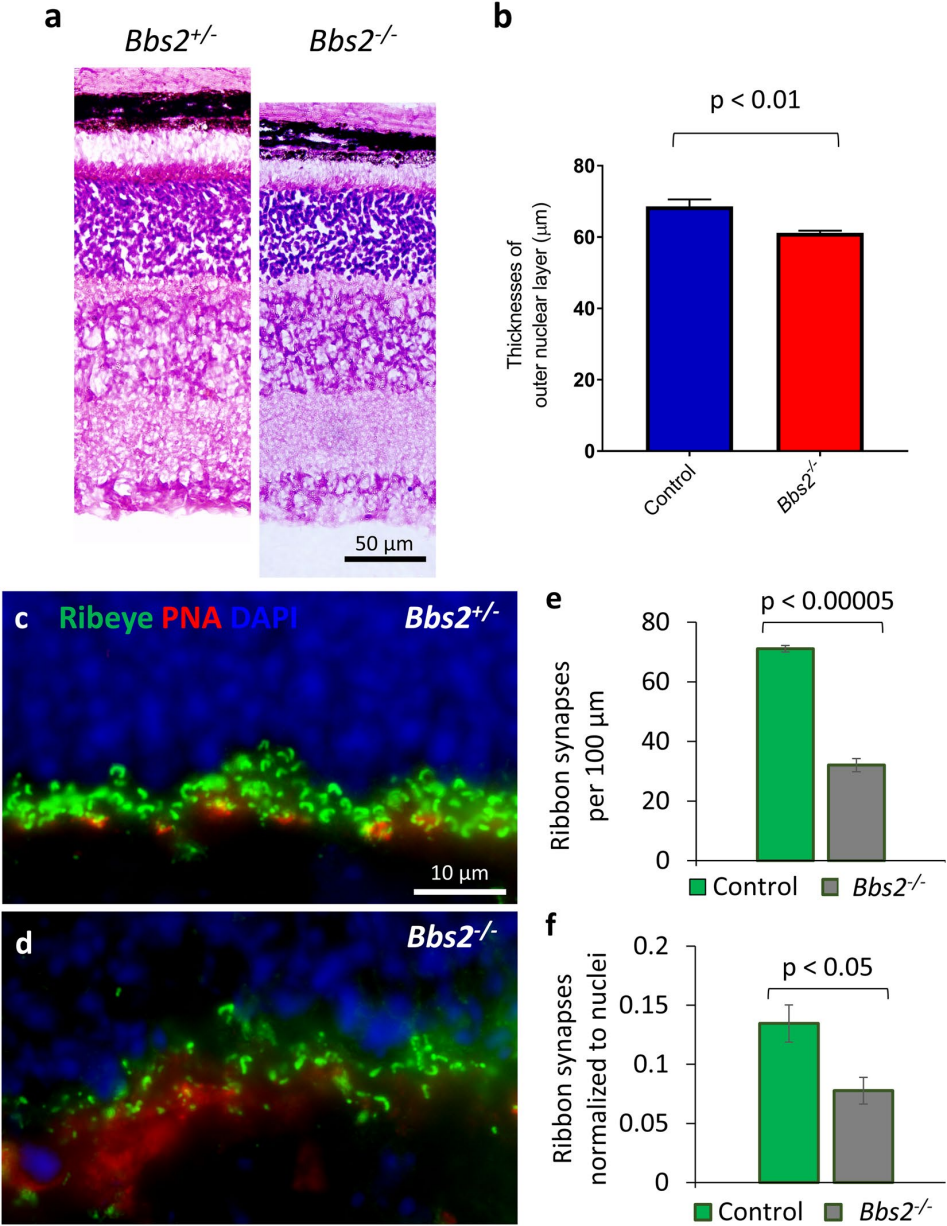
*Bbs2*^−/−^ mice have a reduced number of RIBEYE-positive ribbon synapses at P15-16. At P15-16, *Bbs2*^−/−^ mice have a mild reduction in the thicknesses of their outer nuclear layers (**a,b**). At this age, the number of RIBEYE-positive horse-shoe-shaped ribbon synapses in the outer plexiform layer is reduced in *Bbs2*^−/−^ mice compared to their control littermates (**c,d**; quantification in **e**). After accounting for mild degeneration by normalizing the ribbon synaptic number to photoreceptor nuclei count, the ribbon synaptic density in *Bbs2*^−/−^ mice is 58% of that in their control littermates (**f**).

In order to study ribbon synapses in the outer plexiform layer in BBS retinas, we probed control and *Bbs2*^−/−^ retinas with an anti-CtBP2/RIBEYE antibody. This antibody is frequently used and has been validated to visualize ribbon synapses^31–33^. To capture the formation of ribbon synapses in the retina before major photoreceptor loss, we performed immunohistochemical staining in 15–16 day old mice (postnatal day 15–16, P15-16). At this age, retinal degeneration in *Bbs2*^−/−^ retinas is mild and the thickness of *Bbs2*^−/−^ retinas is 89% of that of their wild type and heterozygous control littermates (Control, 68.6 ± 1.9 μm, n = 7; *Bbs2*^−/−^, 61.2 ± 0.6 μm, n = 6; p = 0.006; Fig. 1a,b). We labeled cone outer segments and pedicles with PNA to visualize the position of outer segments and pedicles. At P15-16, wild type or heterozygous control mice have numerous ribbon synapses neatly confined to the outer plexiform layer (Fig. 1c). In *Bbs2*^−/−^ retinas, the number of ribbon synapses appear greatly reduced, and some ribbon synapses are observed within the outer nuclear layer normally devoid of ribbon synapses (Fig. 1d). To determine whether there is a reduced synaptic density in *Bbs2*^−/−^ retinas, the number of horse-shoe shaped ribbon synapses is quantified and compared to their control littermates. Quantification shows that *Bbs2*^−/−^ retinas have a significantly reduced number of ribbon synapses compared to their wild type or heterozygous control littermates (Control, 71.1 ± 1.1 synapses, n = 6; *Bbs2*^−/−^, 32.1 ± 2.2 synapses, n = 6; p = 1.84 × 10^−5^; Fig. 1e). To account for any photoreceptor cell loss at this stage, the number of ribbon synapses per 100 μm was normalized to the number of photoreceptor nuclei in the outer nuclear layer per 100 μm. After normalization, *Bbs2*^−/−^ retinas have a reduced number of ribbon synapses compared to their wild type or heterozygous control littermates (Control, 0.13 ± 0.02, n = 6; *Bbs2*^−/−^, 0.08 ± 0.01, n = 6; p = 0.010; Fig. 1f). In addition, *Bbs2*^−/−^ mice have an increased number of ectopic ribbon synapses in the outer nuclear layer (Control, 0.3 ± 0.2, n = 5; *Bbs2*^−/−^, 3.2 ± 0.3, n = 5; p = 4.45 × 10^−5^). These results show that *Bbs2*^−/−^ retinas have a reduced ribbon synaptic density that is not accounted for by photoreceptor cell loss. In addition, absence of BBS2 causes some ribbon synapses to ectopically mislocalize to the outer nuclear layer.

**Figure 2:**
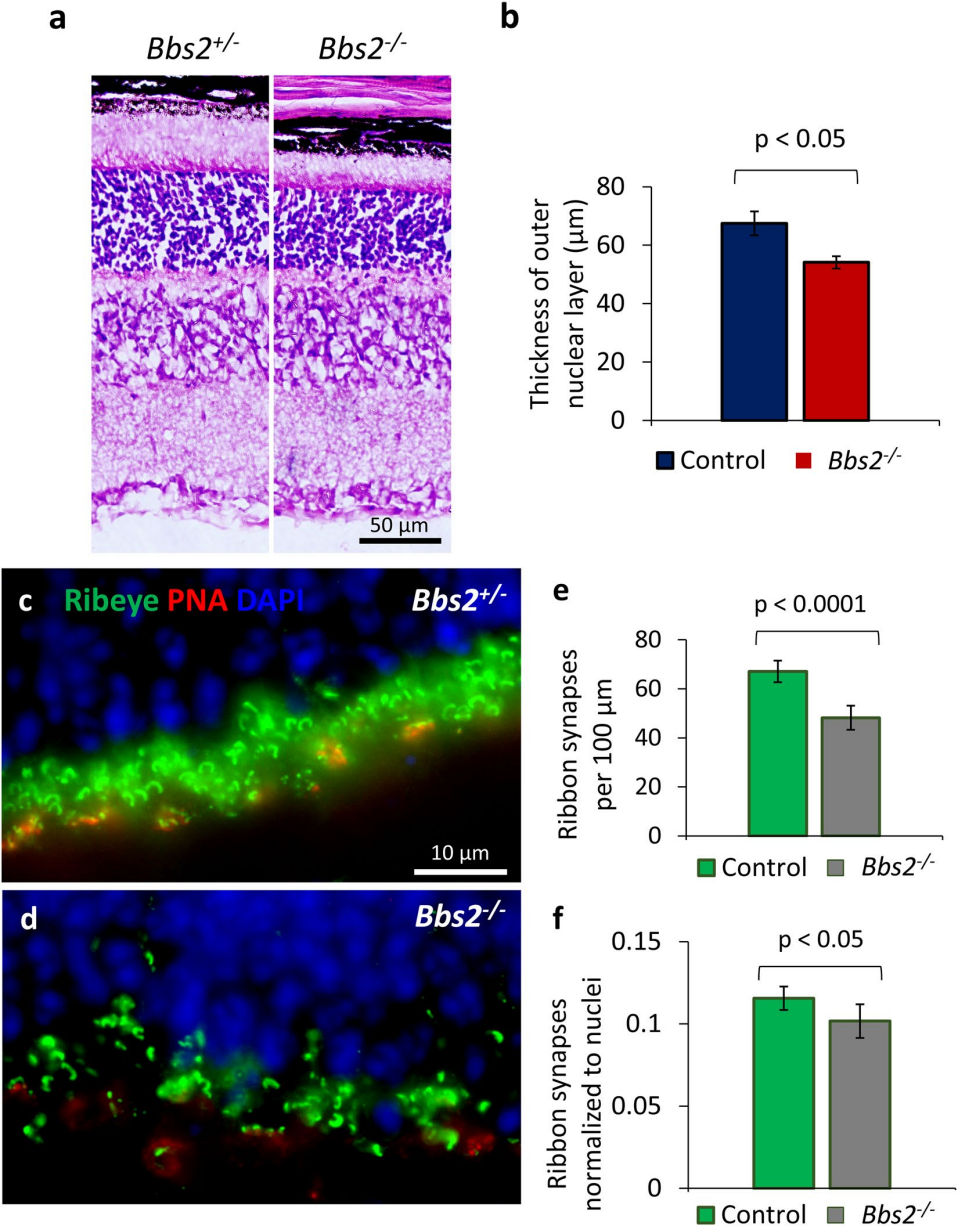
*Bbs2*^−/−^ mice have a reduced number of RIBEYE-positive ribbon synapses at P21. At P21, *Bbs2*^−/−^ mice have a mild reduction in the thicknesses of their outer nuclear layers (**a,b**). At this age, the number of RIBEYE-positive horse-shoe-shaped ribbon synapses in the outer plexiform layer is reduced in *Bbs2*^−/−^ mice compared to their control littermates (**c,d**; quantification in **e**). After accounting for mild degeneration by normalizing the ribbon synaptic number to photoreceptor nuclei count, the ribbon synaptic density in *Bbs2*^−/−^ mice is 88% of that in their control littermates (**f**).

In order to discern whether *Bbs2*^−/−^ retinas develop comparable synaptic density to their control littermates as the retina approaches maturation around the third postnatal week, we examined retinas from 21-day old (P21) mice. At this age, the retinal degeneration in *Bbs2*^−/−^ mice is more advanced compare to that at P15. The thickness of the outer nuclear layers of *Bbs2*^−/−^ mice is approximately 80% of their wild type or heterozygous control littermates (Control, 67.5 ± 4.1 μm, n = 3; *Bbs2*^−/−^, 54.1 ± 2.1 μm, n = 3; p = 0.043; Fig. 2a,b). The number of ribbon synapses in the outer plexiform layer of *Bbs2*^−/−^ retinas is approximately 72% of that of their control littermates (Control, 67.1 ± 4.4 synapses, n = 5; *Bbs2*^−/−^, 48.2 ± 4.9 synapses per 100 μm, n = 5; p = 6.1 × 10^−5^; Fig. 2c–e). It is worth noting that the ribbon synaptic count per 100 μm in the outer plexiform layer in control animals is comparable at P15-16 versus at P21, supporting the notion that the development of ribbon synapses is completed by the end of the second postnatal week in mice^34^. After normalizing to photoreceptor nuclei count, we still observe 12% fewer ribbon synapses in *Bbs2*^−/−^ retinas compared to their control littermates (Control, 0.116 ± 0.007, n = 5; *Bbs2*^−/−^, 0.102 ± 0.010, n = 5; p = 0.038; Fig. 2f). Therefore, *Bbs2*^−/−^ mice have a reduced ribbon synaptic density by the end of retinal synaptogenesis at P15-16, and this defect persists even when the eye reaches maturation at P21.

**Figure 3:**
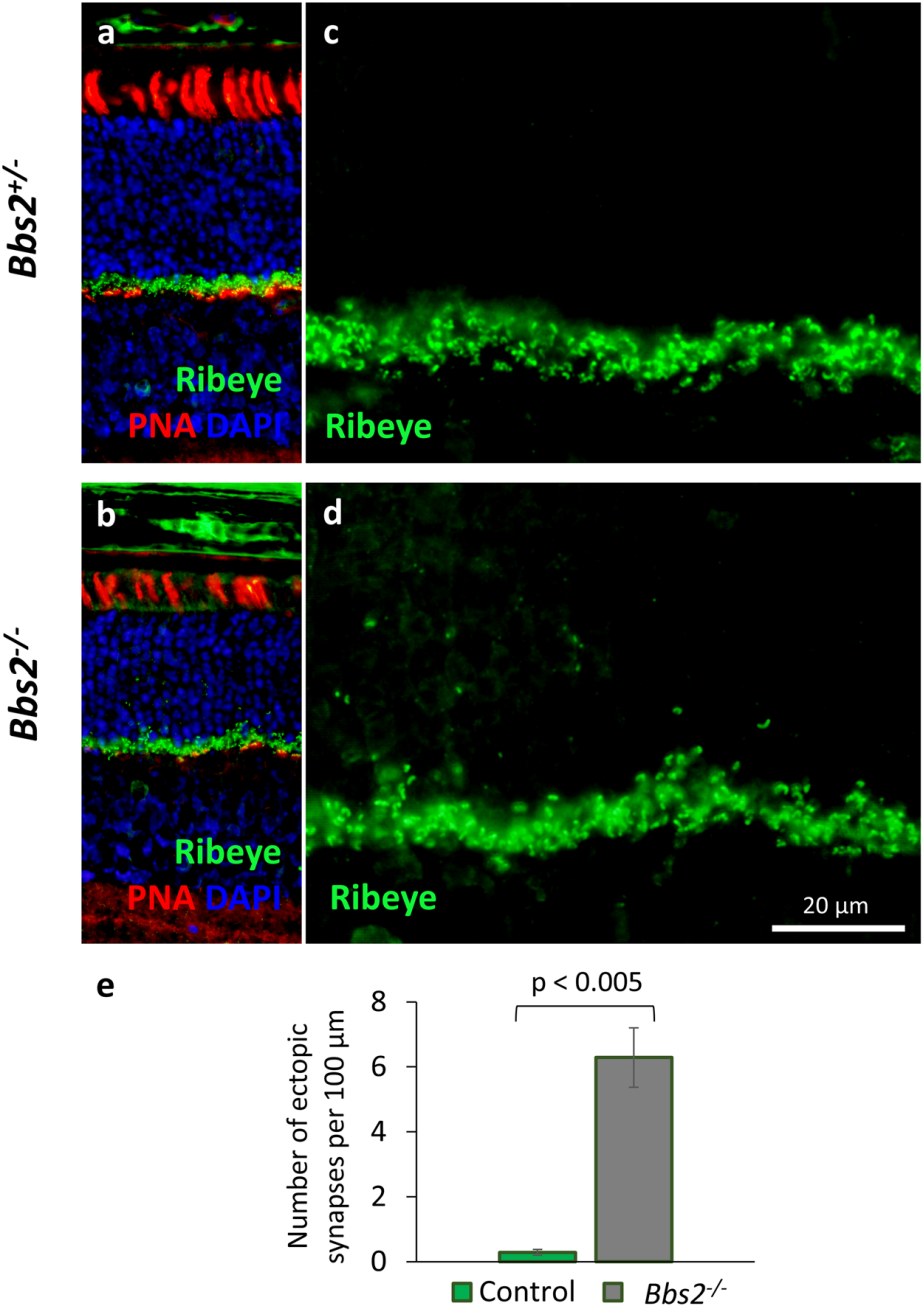
*Bbs2*^−/−^ retinas have an increased number of ectopically localized ribbon synapses. P21 retinas were probed with an anti-RIBEYE antibody to identify ribbon synapses along with biotinylated-PNA to visualize cone outer segments and pedicles (**a,b**). Ectopically localized ribbon synapses within the outer nuclear layer are observed in *Bbs2*^−/−^ mice (**d**), but not in control littermates (**c**). The number of ectopically localized synapses is significantly elevated in *Bbs2*^−/−^ mice (**e**).

At P21, an elevated number of ectopic ribbon synapses is found in the outer nuclear layer of *Bbs2*^−/−^ retinas (Fig. 3). The ribbon synapses in wild type or heterozygous control mice are neatly confined to the outer plexiform layer and are not observed inside the outer nuclear layer (Fig. 3a,c). In contrast, we observed numerous ectopically located ribbon synapses in *Bbs2*^−/−^ retinas within the outer nuclear layer (Fig. 3b,d). Upon quantification, we found that the number of ectopically localized ribbon synapses in the outer nuclear layer is greatly elevated in P21 *Bbs2*^−/−^ retinas (Control, 0.3 ± 0.1 synapses per 100 μm, n = 5; *Bbs2*^−/−^, 6.3 ± 0.9 synapses per 100 μm, n = 5; p = 0.003; Fig. 3e).

### *Bbs7*^−/−^ mice also have a reduced density of ribbon synapses in the outer plexiform layer

**Figure 4:**
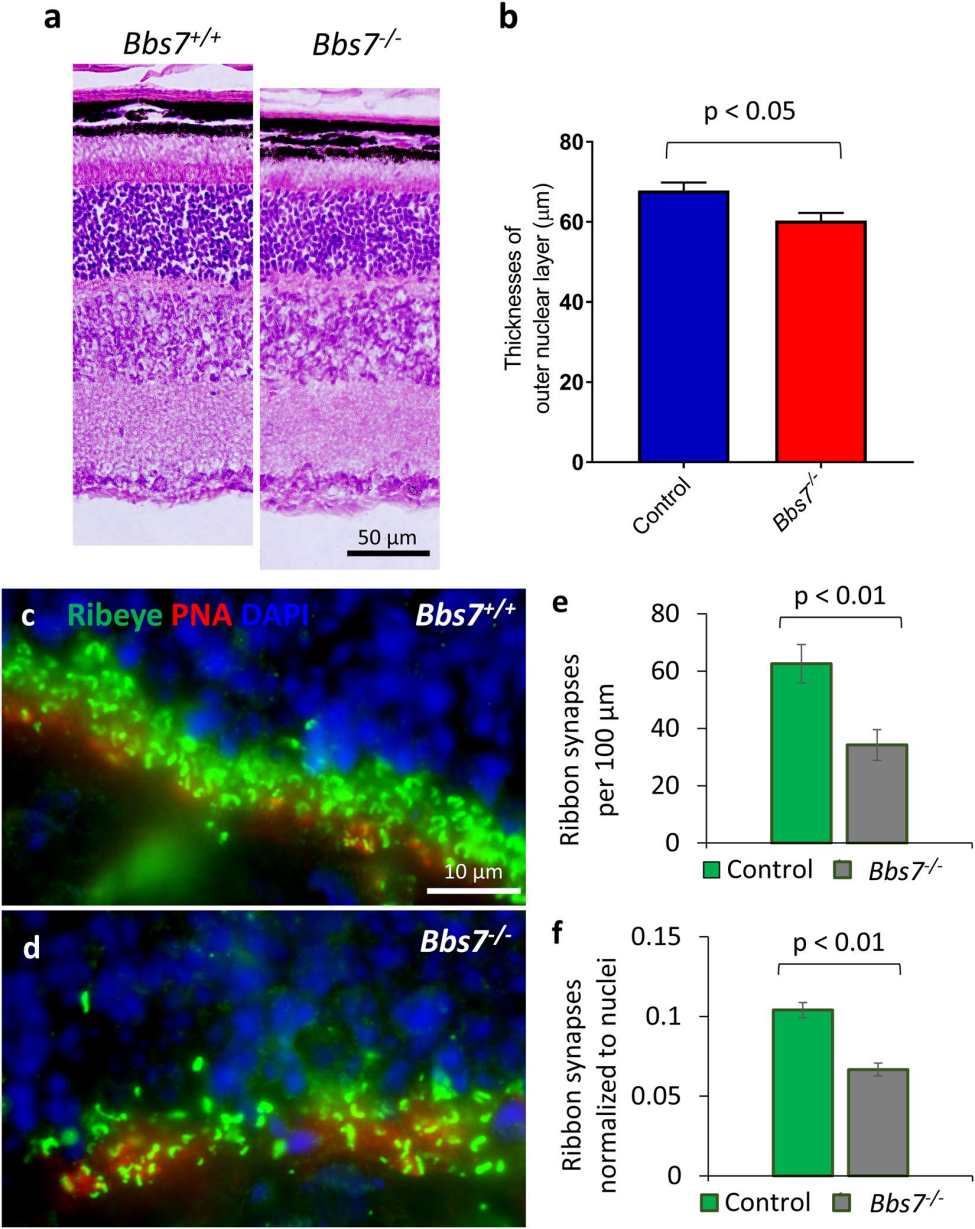
*Bbs7*^−/−^ mice have a reduced number of RIBEYE-positive ribbon synapses at P15-16. At P15-16, *Bbs7*^−/−^ mice have a mild reduction in the thicknesses of their outer nuclear layers (**a,b**). At this age, the number of RIBEYE-positive horse-shoe-shaped ribbon synapses in the outer plexiform layer is reduced in *Bbs7*^−/−^ mice compared to their control littermates (**c,d**; quantification in **e**). After accounting for mild degeneration by normalizing the ribbon synaptic number to photoreceptor nuclei count, the ribbon synaptic density in *Bbs7*^−/−^ mice is 64% of that in their control littermates (**f**).

The BBSome is a protein complex consisting of BBS1, BBS2, BBS4, BBS5, BBS7, BBS8, BBS9 and BBS18^1–9^, and a functional BBSome requires all of its components^6,35^. In order to determine whether the reduced synaptic density phenotype is caused by the loss of BBSome function or is specific to loss of BBS2, we repeated the analysis in P15-16 *Bbs7*^−/−^ mice, another BBS mouse model in which the deletion of BBS7 causes the lack of BBSome function^13^. Similar to that observed in *Bbs2*^−/−^ mice, there is a mild reduction in the thickness of the outer nuclear layer in *Bbs7*^−/−^ mice (Control, 67.9 ± 2.0 μm, n = 5; *Bbs7*^−/−^, 60.3 ± 1.9 μm, n = 3; p = 0.047; Fig. 4a,b). Quantification reveals that control mice have 62.6 ± 6.7 ribbon synapses per 100 μm, whereas their *Bbs7*^−/−^ littermates have 34.2 ± 5.4 ribbon synapses, a 45% reduction (Control, n = 3; *Bbs7*^−/−^, n = 3; p = 0.006; Fig. 4e). After normalizing for photoreceptor nuclei count, we still observe 36% fewer ribbon synapses in *Bbs7*^−/−^ retinas compared to their control littermates (Control, 0.10 ± 0.005, n = 3; *Bbs7*^−/−^, 0.067 ± 0.004, n = 3; p = 0.007; Fig. 4f). In addition, *Bbs7*^−/−^ mice have an increased number of ectopic synapses in the outer nuclear layer (Control, 0.2 ± 0.1 synapses, n = 3; *Bbs7*^−/−^, 4.6 ± 0.8 synapses, n = 3; p = 0.0045). Since *Bbs2*^−/−^ and *Bbs7*^−/−^ mice share the phenotypes of reduced ribbon synaptic densities and ectopic synapses, these phenotypes are likely caused by the lack of BBSome function in both mouse models, and are not specific to BBS2 or BBS7.

### Absence of BBSome function interferes with normal synaptogenesis

**Figure 5:**
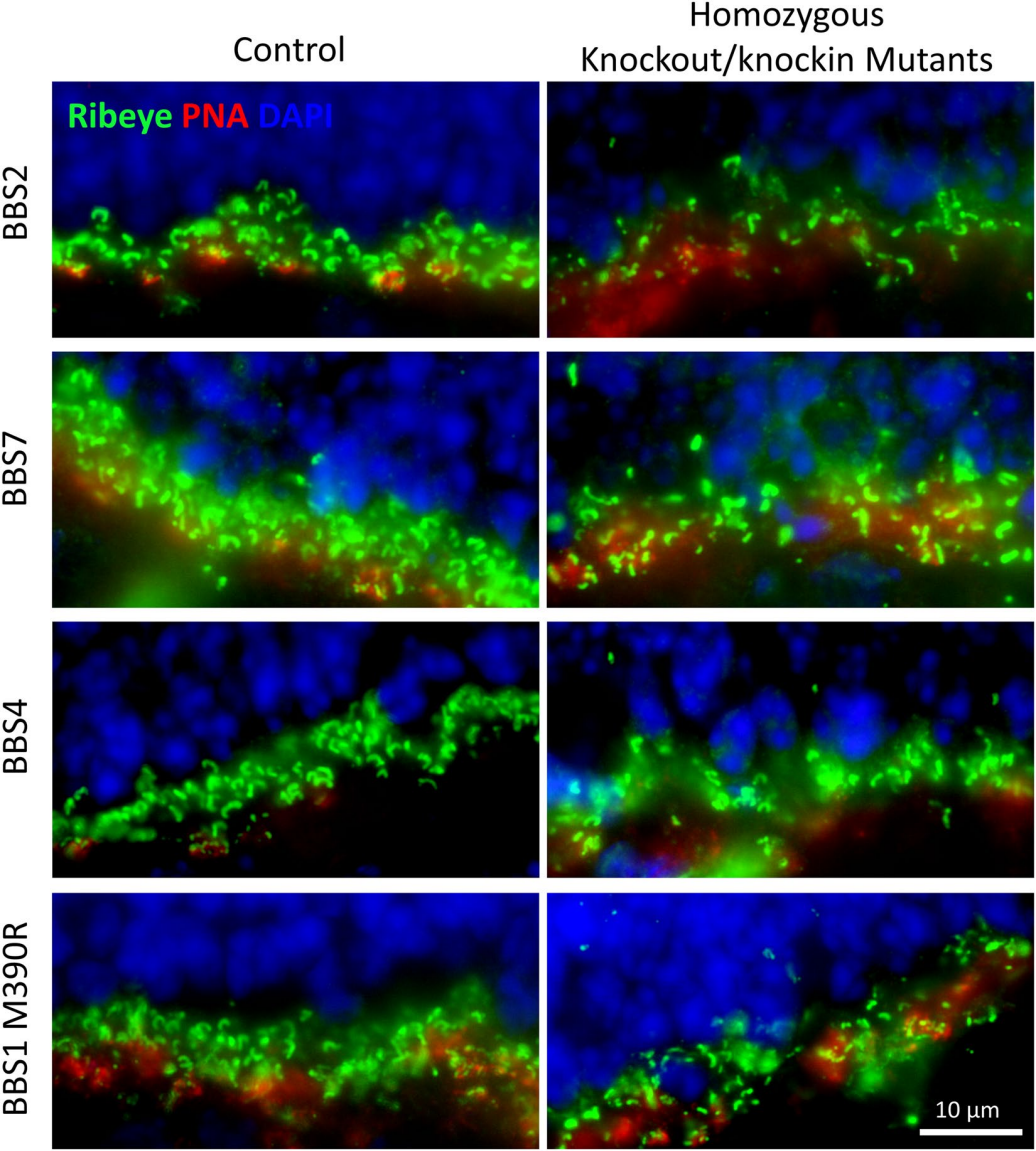
Mutant mouse models of the BBSome have disorganized and reduced number of ribbon synapses as early as P15. P15-16 retinas from *Bbs2*^−/−^, *Bbs7*^−/−^, *Bbs4*^−/−^, *Bbs1*^*M390R/M390R*^ and their respective control littermates were probed with an anti-CtBP2/RIBEYE antibody and biotinylated-PNA to visualize ribbon synapses in the outer plexiform layer. The images are oriented such that the outer nuclear layer is above, and the inner nuclear layer is below the ribbon synapses.

We also probed the retinas of *Bbs4*^−/−^ and *Bbs1*^*M390R/M390R*^ mutant mice at P15-P16 with the anti-CtBP2/RIBEYE antibody and biotinylated-PNA to determine whether these phenotypes are observed in other mouse models with mutated components of the BBSome. Similar to the observation in *Bbs2*^−/−^ and *Bbs7*^−/−^ retinas, the density of ribbon synapses in the outer plexiform layer in *Bbs4*^−/−^ and *Bbs1*^*M390R/M390R*^ retinas appears greatly reduced compared to their control littermates, and some synapses are mislocalized (Fig. 5). Since all of the mutant mouse models of the BBSome we examined share the phenotype of reduced and ectopic ribbon synapse formation, we conclude that lack of BBSome function interferes with normal synaptogenesis, reduces ribbon synaptic density in the outer plexiform layer in the retina, and causes ectopic localization of ribbon synapses.

### Some ectopic ribbon synapses proximal to the outer plexiform layer are associated with intruding bipolar cell processes

**Figure 6:**
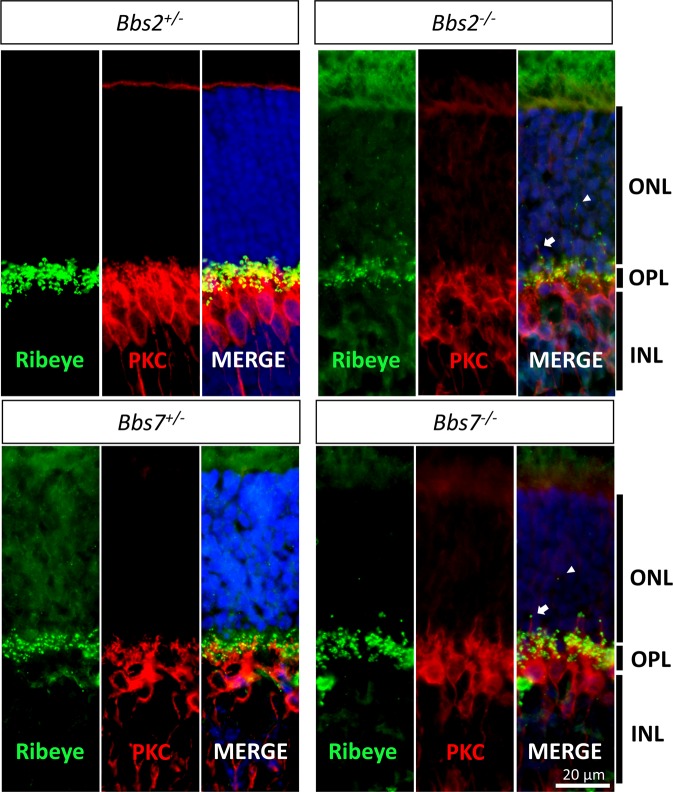
Bipolar cell processes decorated with ribbon synapses intrude into the outer nuclear layer in BBS mutant retinas. P21 retinas from *Bbs2*^−/−^, *Bbs7*^−/−^ and their control littermates were probed with anti-PKCα and anti-CtBP2/RIBEYE antibodies to visualize bipolar cells and ribbon synapses. In control retinas, PKCα-positive bipolar cell dendrites appear as nodules that contact photoreceptor ribbon synapses in an organized fashion in the outer plexiform layer. In *Bbs2*^−/−^ and *Bbs7*^−/−^ retinas, bipolar cell processes decorated with ribbon synapses are sometimes observed extending into the outer nuclear layer. ONL: outer nuclear layer; OPL: outer plexiform layer; INL: inner nuclear layer.

Ectopic ribbon synapses could be caused by aberrant process extension of bipolar and/or horizontal cells into the outer nuclear layer. In order to determine whether intruding bipolar cell processes contribute to the ectopic ribbon synapse phenotype, we probed P21 *Bbs2*^−/−^ and *Bbs7*^−/−^ retinas with an anti-PKCα antibody along with the anti-CtBP2/RIBEYE antibody to visualize the morphology of bipolar cells and ribbon synapses. In both control and knockout retinas, the anti-PKCα antibody recognizes numerous bipolar cell bodies situated in the inner nuclear layer possessing long, vertical processes extending towards the ganglion cells (Fig. 6). In control retinas, bipolar cell dendrites appear as knob-like protrusions enveloped by the photoreceptor ribbon synapses, and these bipolar-photoreceptor contacts are orderly confined to the outer plexiform layer. In *Bbs2*^−/−^ and *Bbs7*^−/−^ retinas, however, some bipolar cell processes decorated with ribbon synapses intrude into the outer nuclear layer (Fig. 6, arrows). However, none of the aberrant bipolar cell processes intrude beyond a third of the thickness of the outer nuclear layer, and there are ectopic ribbon synapses observed beyond this distance within the outer nuclear layer that do not appear to be associated with these bipolar cell processes (Fig. 6, arrowheads). Therefore, aberrant extension of bipolar cell processes may account for some ectopic synapses located more proximal to the outer plexiform layer in BBS mutant retinas. Those ectopic synapses observed distant from the outer plexiform layer are not accounted for by intruding bipolar cell processes.

### Ectopic ribbon synapses in *Bbs2*^−/−^, *Bbs7*^−/−^, and *Bbs8*^−/−^ mice are associated with invading axonal processes of horizontal cells

**Figure 7:**
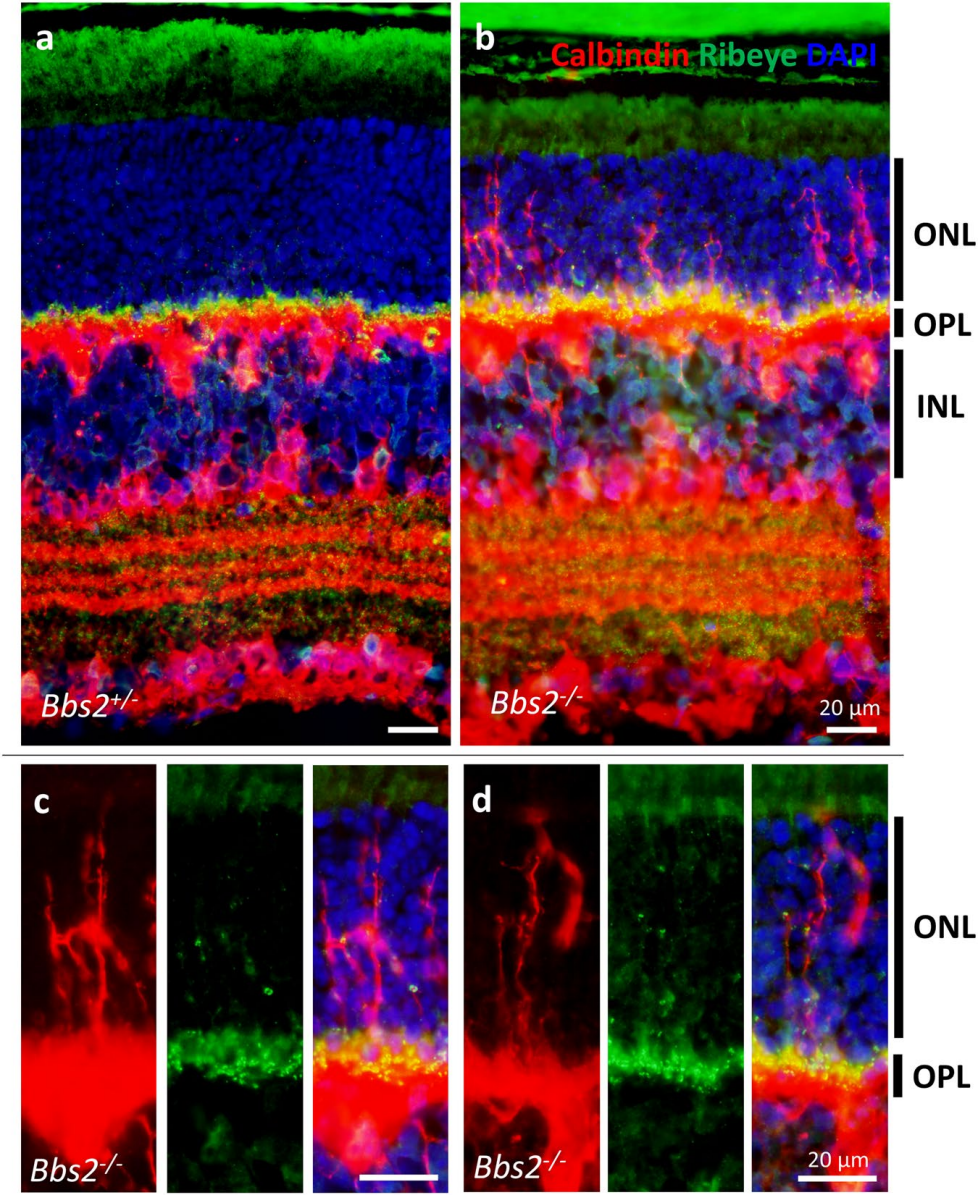
Aberrant horizontal cell processes intrude into the outer nuclear layer in *Bbs2*^−/−^ retinas. P21 retinas from *Bbs2*^−/−^ and control littermates were probed with anti-calbindin and anti-CtBP2/RIBEYE antibodies. In control retinas (**a**), the outer nuclear layer lacks immunoreactive signals from both antibodies. In *Bbs2*^−/−^ retinas (**b–d**), calbindin-positive horizontal cell processes decorated by ribbon synapses are observed in the outer nuclear layer, frequently spanning the entire thickness of the outer nuclear layer. ONL: outer nuclear layer; OPL: outer plexiform layer; INL: inner nuclear layer.

**Figure 8:**
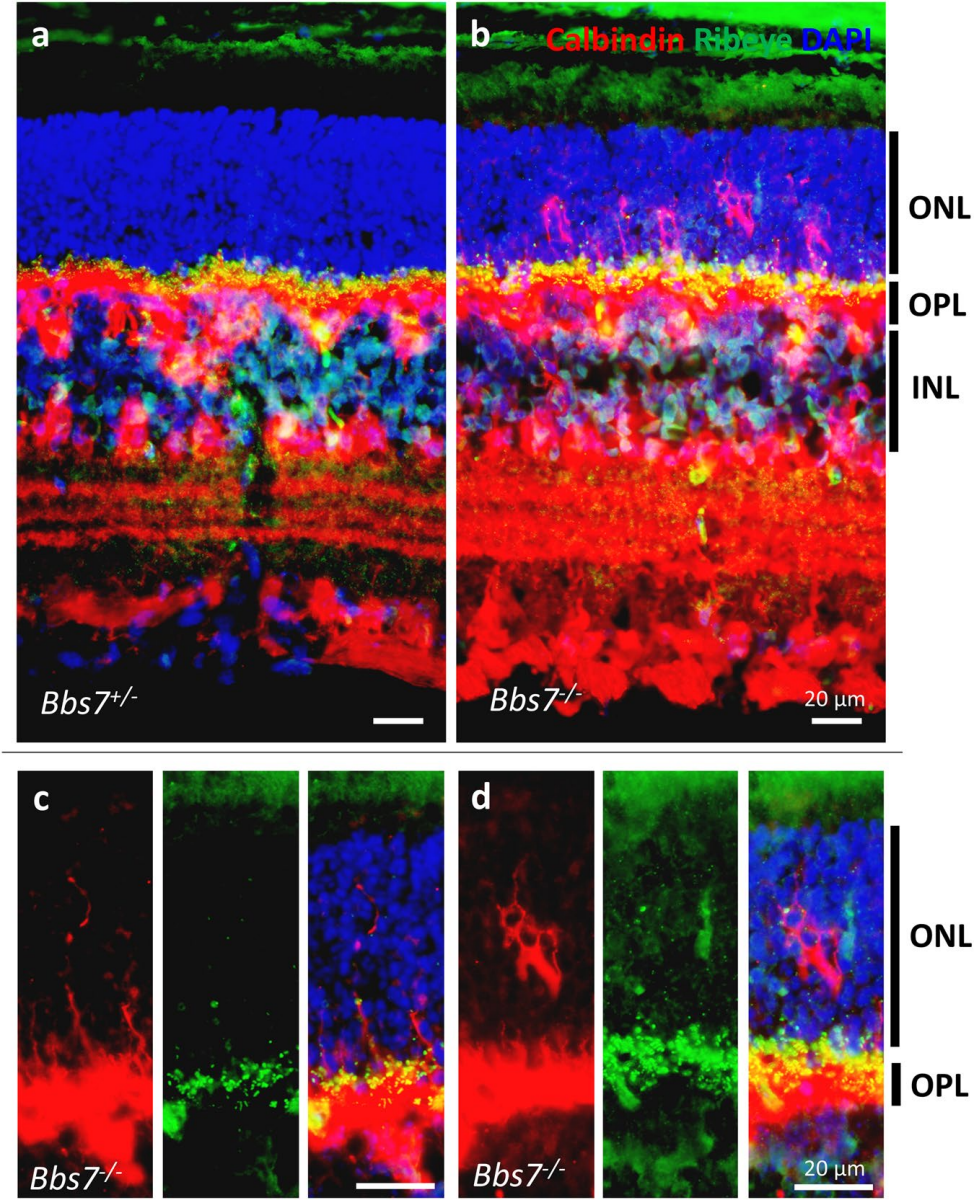
Aberrant horizontal cell processes intrude into the outer nuclear layer in *Bbs7*^−/−^ retinas. P21 retinas from *Bbs7*^−/−^ and control littermates were probed with anti-calbindin and anti-CtBP2/RIBEYE antibodies. In control retinas (**a**), the outer nuclear layer lacks immunoreactive signals from both antibodies. In *Bbs7*^−/−^ retinas (b, c, d), calbindin-positive horizontal cell processes decorated by ribbon synapses are observed in the outer nuclear layer, frequently spanning the entire thickness of the outer nuclear layer. ONL: outer nuclear layer; OPL: outer plexiform layer; INL: inner nuclear layer.

**Figure 9:**
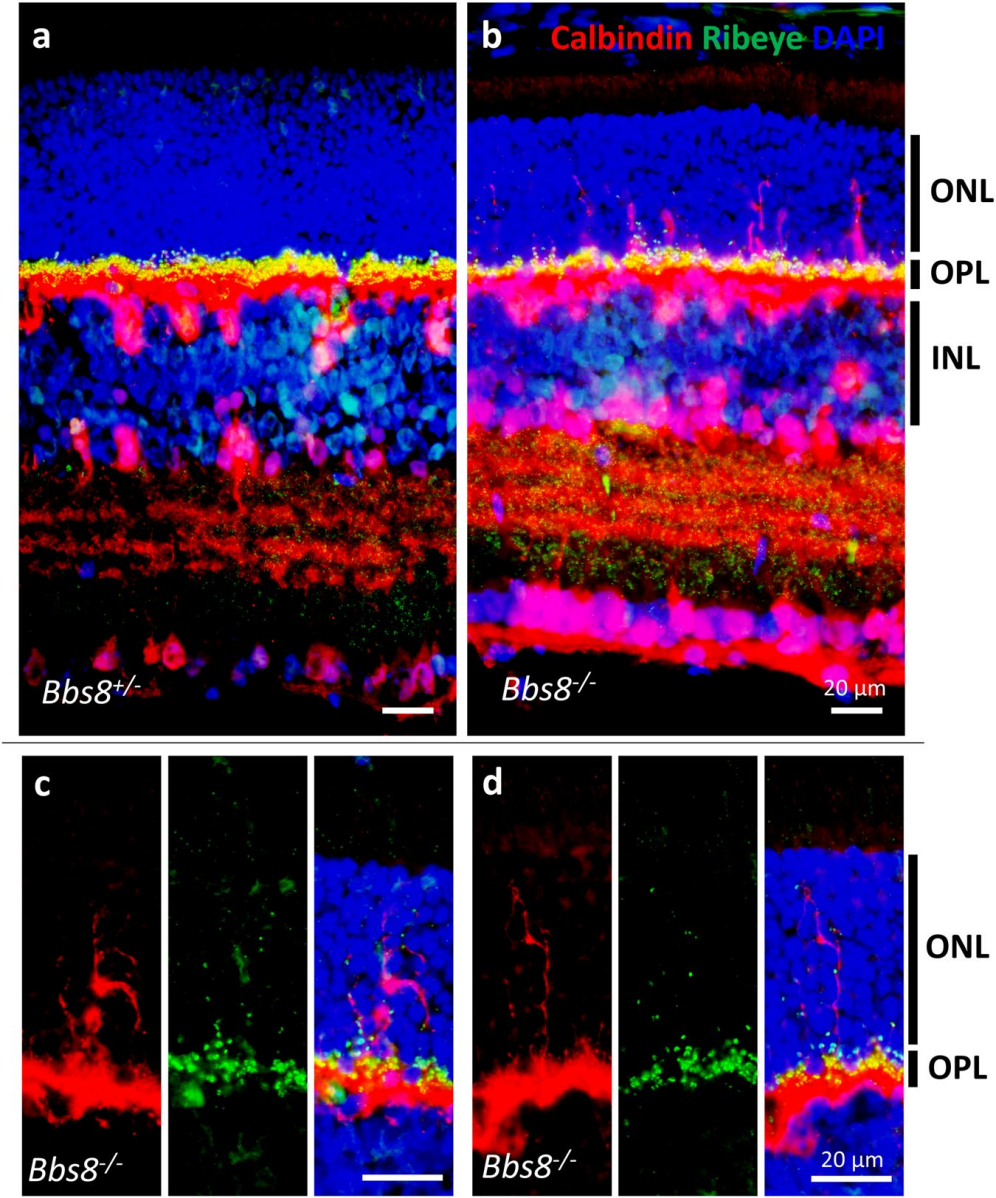
Aberrant horizontal cell processes intrude into the outer nuclear layer in *Bbs8*^−/−^ retinas. P21 retinas from *Bbs8*^−/−^ and control littermates were probed with anti-calbindin and anti-CtBP2/RIBEYE antibodies. In control retinas (**a**), the outer nuclear layer lacks immunoreactive signals from both antibodies. In *Bbs8*^−/−^ retinas (**b–d**), calbindin-positive horizontal cell processes decorated by ribbon synapses are observed in the outer nuclear layer, frequently spanning the entire thickness of the outer nuclear layer. ONL: outer nuclear layer; OPL: outer plexiform layer; INL: inner nuclear layer.

Furthermore, we probed P21 control and *Bbs2*^−/−^ retinas with an anti-calbindin antibody together with the anti-CtBP2/RIBEYE antibody in order to visualize the morphology of horizontal cells and ribbon synapses. In wild type or heterozygous control animals, horizontal cell bodies are found in the upper portion of the inner nuclear layer, and the outer nuclear layer is devoid of any intruding horizontal cell processes (Fig. 7a). It is worth noting that some amacrine cells and ganglion cells also stain positive for calbindin as previously observed^36^. In contrast to their littermate controls, numerous calbindin-positive processes are observed within the outer nuclear layer in *Bbs2*^−/−^ retinas (Fig. 7b–d). These processes are likely horizontal cell processes, since they are contiguous with horizontal cell bodies. Some of these processes extend across the entire outer nuclear layer, reaching the outer limiting membrane (Fig. 7c,d). Occasionally, it appears that an entire horizontal cell has migrated into the outer nuclear layer, although the great majority of the horizontal cell bodies are still confined to their normal positions in the inner nuclear layer. These aberrant horizontal cell processes are decorated with ribbon synapses in *Bbs2*^−/−^ retinas (Fig. 7c,d). Ectopic ribbon synapses observed at greater than one third of the thickness of the outer nuclear layer, beyond the reach of the intruding bipolar cell processes, are associated with the processes of these horizontal cells. Similar observations are made using P21 *Bbs7*^−/−^ retinas (Fig. 8) and *Bbs8*^−/−^ retinas (Fig. 9), suggesting that this phenomenon is not specific to loss of BBS2, but due to the loss of BBSome function in these mice. Together, these results suggest that horizontal cell processes invade the outer nuclear layer in BBS mutant mice, sometimes spanning the entire thickness of the outer nuclear layer. These intruding processes are frequently associated with ectopic ribbon synapses and may contribute to the formation of the ectopic ribbon synapses in the outer nuclear layer.

**Figure 10:**
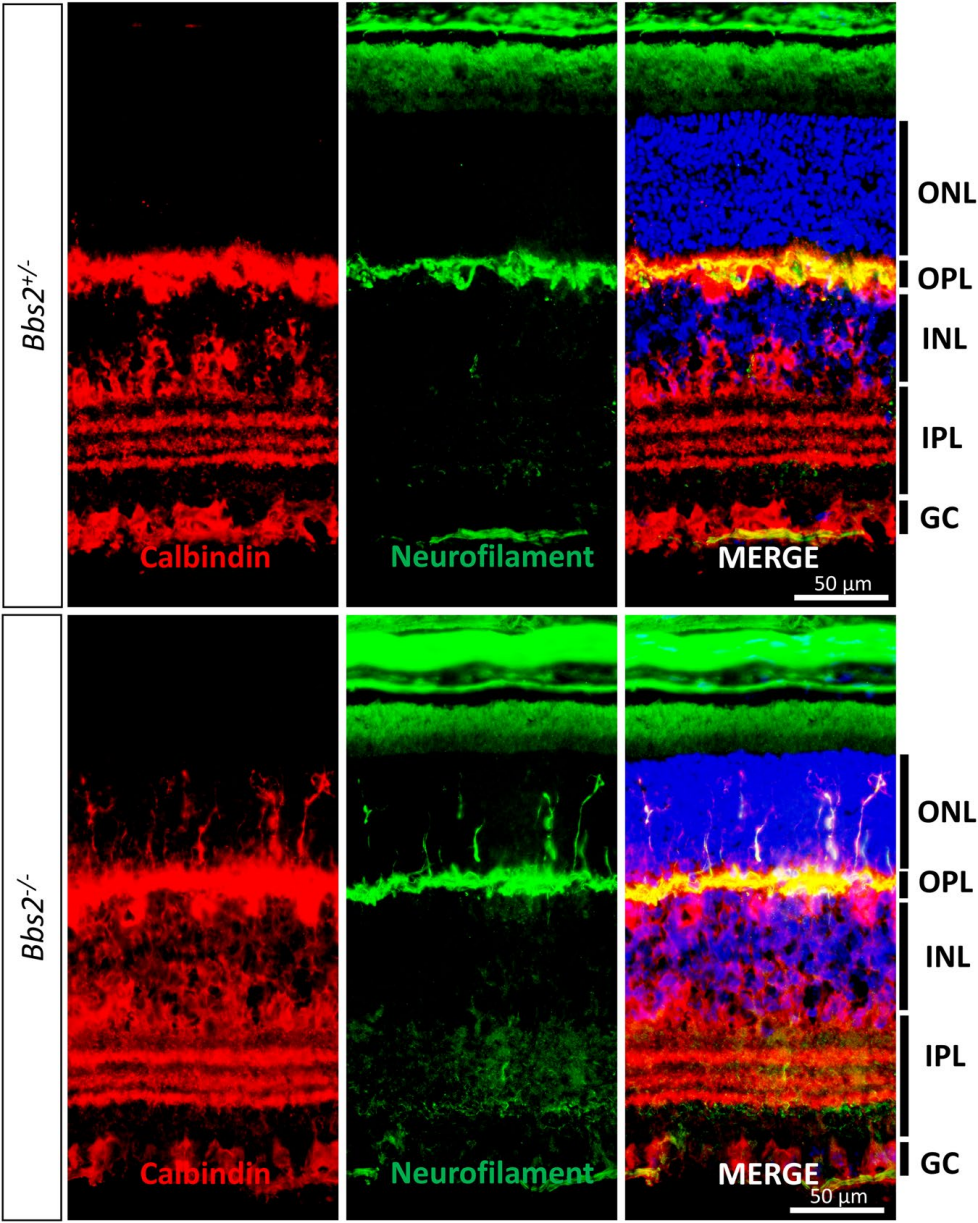
Intruding horizontal cell processes in the outer nuclear layers of BBS mutant mice are positive for axonal marker neurofilament. P21 retinas from *Bbs2*^−/−^ and control littermates were probed with anti-calbindin and anti-neurofilament antibodies. In control retinas, the immunoreactive signal from the axonal marker anti-neurofilament antibody overlaps with the signal from anti-calbindin antibody. In *Bbs2*^−/−^ retinas, aberrant horizontal cell processes in the outer nuclear layer that are calbindin-positive are also neurofilament-positive. ONL: outer nuclear layer; OPL: outer plexiform layer; INL: inner nuclear layer; IPL: inner plexiform layer; GC: ganglion cells.

In order to determine whether these intruding processes are axonal processes of horizontal cells, we probed the P21 control and *Bbs2*^−/−^ retinal sections with an anti-neurofilament antibody targeting the neurofilament medium chain subunit along with the anti-calbindin antibody. Neurofilaments are intermediate filaments that are enriched in axons^37^. Axonal terminals of horizontal cells are neurofilament-positive^38^. In control retinas, the anti-neurofilament antibody recognizes filamentous structures in the outer plexiform layer that are also immunoreactive to the anti-calbindin antibody (Fig. 10, upper panels). These neurofilament-positive processes of horizontal cells are confined to the outer plexiform layer. In *Bbs2*^−/−^ retinas, calbindin-positive horizontal cell processes that invade into the outer nuclear layer are also positive for neurofilament (Fig. 10, lower panels). Therefore, in BBS mutant mice, those processes that aberrantly intrude into the outer nuclear layer are axonal processes of horizontal cells.

### Evidence for a cell-autonomous role of the BBSome in horizontal cells

**Figure 11:**
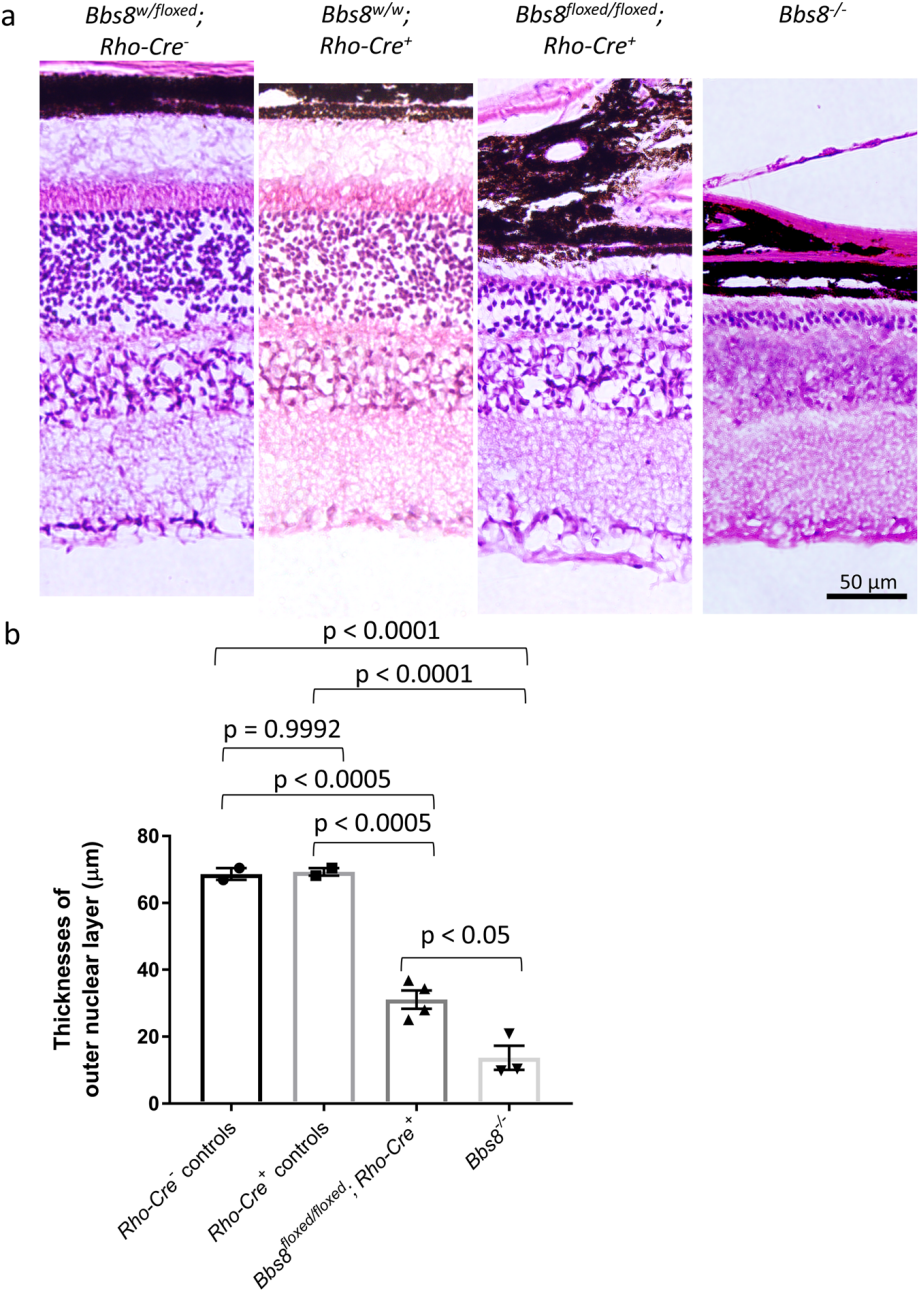
Photoreceptor-specific inactivation of BBS8 via *Cre*-mediated recombination driven by the rhodopsin promoter causes retinal degeneration. Histological sections of the retinas from 6–7-month-old *Bbs8*^*w/floxed*^*; Rho-Cre*^*-*^, *Bbs8*^*w/w*^*; Rho-Cre*^+^, *Bbs8*^*floxed/floxed*^*; Rho-Cre*^+^, and *Bbs8*^−/−^ mice (**a**). Thicknesses of the outer nuclear layers were quantified by an observer masked to genotypes, and compared by one-way ANOVA with post-hoc Tukey’s test (**b**).

Since the intrusion of bipolar and/or horizontal cell processes into the outer nuclear layer is also observed in non-BBS mutant mouse models that have retinal degeneration, it is not clear whether it is a mere consequence of photoreceptor degeneration or whether BBS genes play a role in regulating the process extension of inner neurons such as horizontal cells. Therefore, using a mouse line where the expression of CRE recombinase is driven by the rhodopsin promoter^30^, we generated *Bbs8*^*floxed/floxed*^*; Rho-Cre*^+^ mice, in which BBS8 proteins are specifically deleted in photoreceptor cells, to investigate whether retinal degeneration triggered by the loss of BBSome function in photoreceptors alone causes the intrusion of horizontal cell processes into the outer nuclear layer. We have previously shown that the absence of BBS8 in the eye causes the loss of the complete BBSome complex and BBSome function^20^. In *Bbs8*^*floxed/floxed*^*; Rho-Cre*^+^ mice, we observe retinal degeneration over time. At 6–7 months of age, when 80% of photoreceptors are lost in *Bbs8* congenital knockout mice (*Bbs8*^−/−^), the thickness of the outer nuclear layer in *Bbs8*^*floxed/floxed*^*; Rho-Cre*^+^ mice is reduced by 55% compared to that in *Bbs8*^*w/floxed*^*; Rho-Cre*^*−*^ mice (Fig. 11). The thickness of the outer nuclear layer in 6–7-month-old *Bbs8*^*w/w*^ *or Bbs8*^*w/floxed*^*; Rho-Cre*^+^ mice with at least one copy of the wild type *Bbs8* gene is not statistically different from that in *Bbs8*^*w/floxed*^*; Rho-Cre*^*−*^ mice (p = 0.999), indicating that the presence of CRE did not cause adverse effects in photoreceptor cells (Fig. 11).

**Figure 12:**
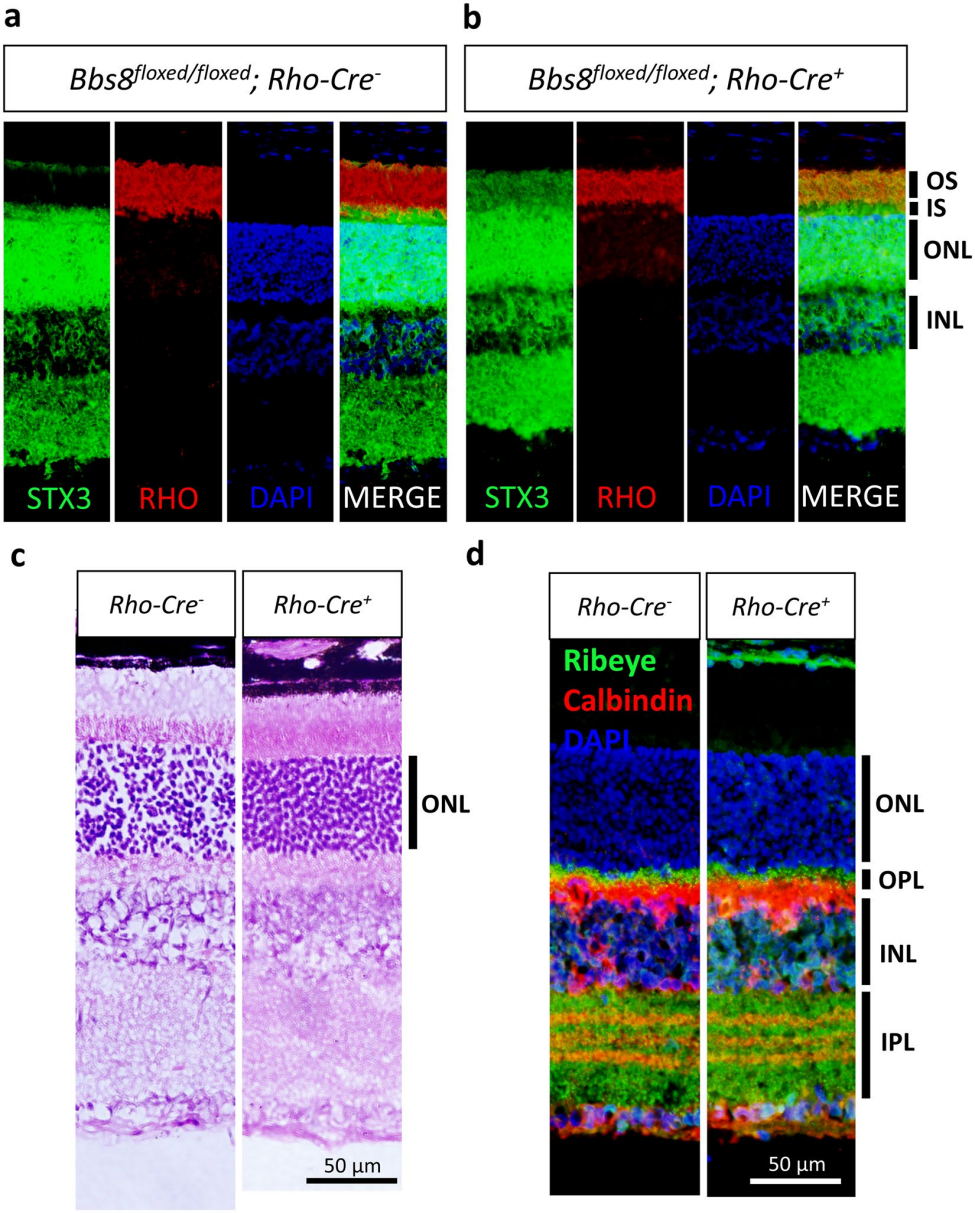
One-month-old *Bbs8*^*floxed/floxed*^*; Rho-Cre*^+^ mice lose inner segment retention of syntaxin-3 (STX3). STX3, a vesicle fusion protein normally confined to the photoreceptor inner segment (**a**), mislocalizes to the outer segment in one-month-old *Bbs8*^*floxed/floxed*^*; Rho-Cre*^+^ mice (**b**), indicating the loss of BBSome function in these photoreceptors. At this age, the thicknesses of the outer nuclear layers are not significantly different between *Rho-Cre*^*-*^ and *Rho-Cre*^+^ mice (**c**). Intrusion of horizontal cell processes into the outer nuclear layer is not observed in 1-month-old *Bbs8*^*floxed/floxed*^*; Rho-Cre*^+^ mice (**d**).

We have previously shown that the absence of BBSome function in photoreceptors causes the loss of inner segment retention of syntaxin-3 (STX3), a vesicle fusion protein^20^. At 1 month of age, STX3, which is normally confined to the photoreceptor inner segments (Fig. 12a), is present in photoreceptor outer segments in *Bbs8*^*floxed/floxed*^*; Rho-Cre*^+^ mice (Fig. 12b), indicating the loss of BBSome function in the photoreceptors of these mice. At this age, the thicknesses of the outer nuclear layers in *Bbs8*^*floxed/floxed*^*; Rho-Cre*^+^ mice appear slightly thinner than that in *Bbs8*^*floxed/floxed*^*; Rho-Cre*^*−*^ mice, but the difference is not yet statistically significant (*Bbs8*^*floxed/floxed*^*; Rho-Cre*^*−*^, 63.0 ± 1.7 μm, n = 5; *Bbs8*^*floxed/floxed*^*; Rho-Cre*^+^, 58.3 ± 2.2 μm, n = 5; p = 0.13; Fig. 12c), indicating that the process of photoreceptor degeneration has just begun. Ectopic synapses or wandering horizontal cell processes are not observed in the outer nuclear layers of 1-month-old *Bbs8*^*floxed/floxed*^*; Rho-Cre*^+^ mice (Fig. 12d).

**Figure 13:**
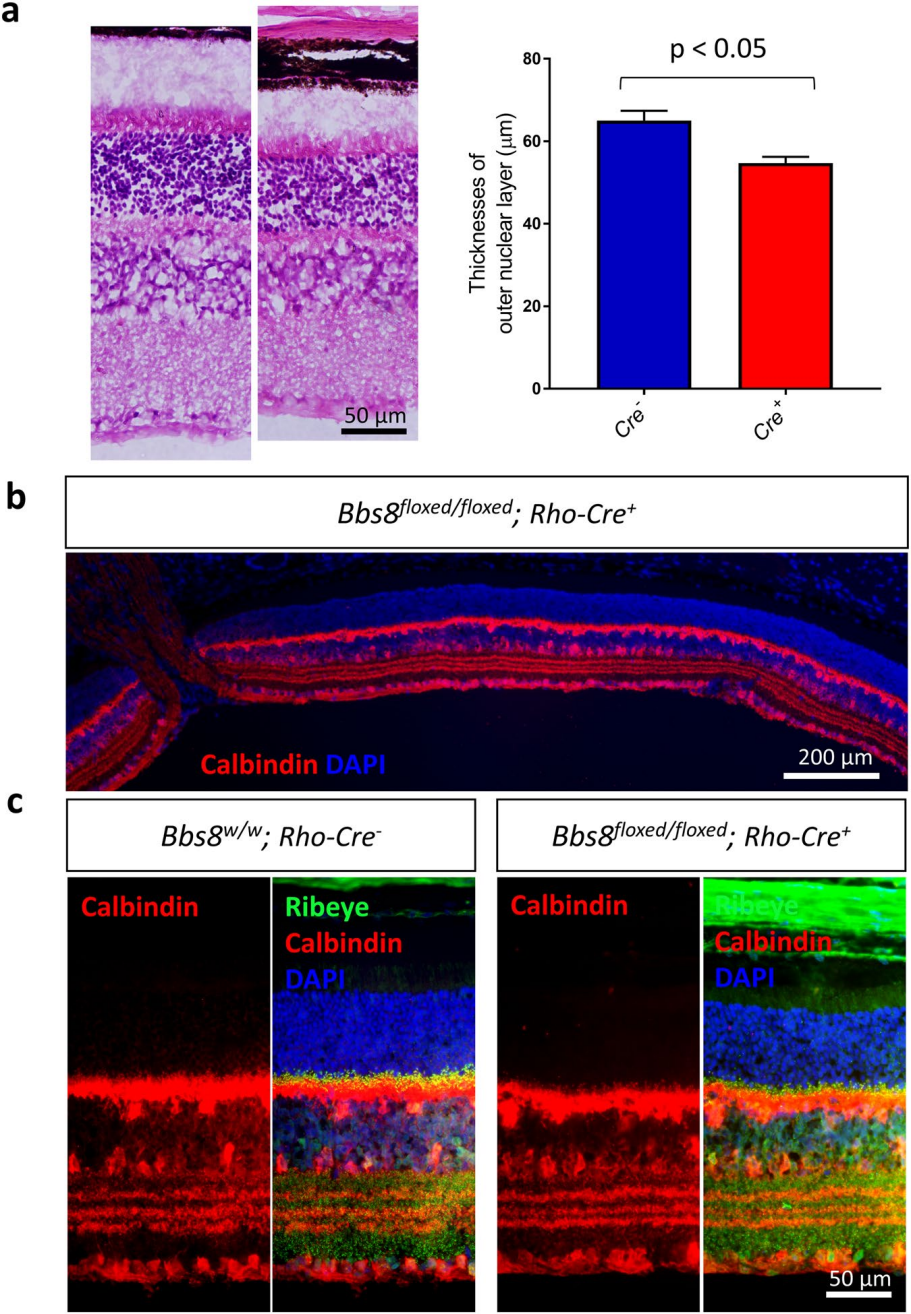
Aberrant intrusion of horizontal cell processes into the outer nuclear layer is not observed in 3-month-old *Bbs8*^*floxed/floxed*^*; Rho-Cre*^+^ mice with retinal degeneration. Three-month-old *Bbs8*^*floxed/floxed*^*; Rho-Cre*^+^ mice have a statistically significant reduction in the thicknesses of their outer nuclear layers (**a**). However, despite retinal degeneration, the outer nuclear layers of *Bbs8*^*floxed/floxed*^*; Rho-Cre*^+^ mice are largely devoid of aberrantly intruding horizontal cell processes (**b,c**).

If the wandering processes of horizontal cells are a consequence of retinal degeneration, then these intruding horizontal cell processes would be observed in *Bbs8*^*floxed/floxed*^*; Rho-Cre*^+^ mice once retinal degeneration is underway. Therefore, we probed 3-month-old *Bbs8*^*floxed/floxed*^*; Rho-Cre*^+^ retinas with the anti-calbindin antibody and the anti-CtBP2/RIBEYE antibody to visualize horizontal cells. At 3 months of age, there is a statistically significant reduction in the thicknesses of the outer nuclear layers in *Bbs8*^*floxed/floxed*^*; Rho-Cre*^+^ mice (*Bbs8*^*w/w*^*; Rho-Cre*^−^, 65.1 ± 2.3 μm, n = 13; *Bbs8*^*floxed/floxed*^*; Rho-Cre*^+^, 54.7 ± 1.5 μm, n = 5; p = 0.018; Fig. 13a). However, intrusion of horizontal cell processes into the outer nuclear layer is rarely observed in 3-month-old *Bbs8*^*floxed/floxed*^*; Rho-Cre*^+^ mice (Fig. 13b,c), unlike in P21 congenital BBS knockout mice.

**Figure 14:**
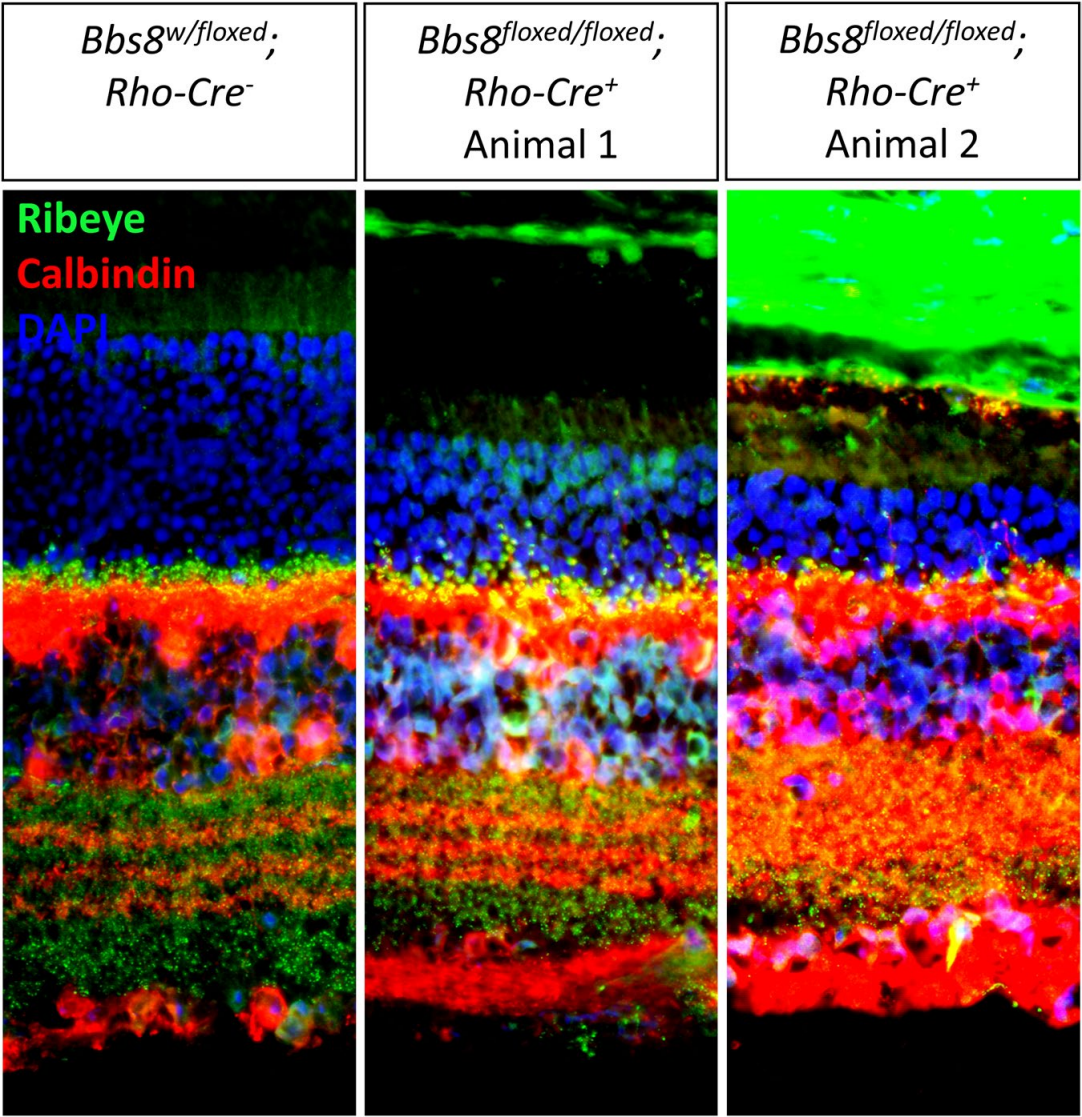
Intrusion of horizontal cell processes into the outer nuclear layer in 6-month-old *Bbs8*^*floxed/floxed*^*; Rho-Cre*^+^ mice induced by retinal degeneration does not recapitulate the phenotypic severity observed in P21 BBS mutant mice. At 6–7 months, some horizontal cell processes decorated with ribbon synapses are observed within the outer nuclear layer in *Bbs8*^*floxed/floxed*^*; Rho-Cre*^+^ mice.

In 6-month-old *Bbs8*^*floxed/floxed*^*; Rho-Cre*^+^ mice with more than 50% photoreceptor loss, we do observe some intrusion of horizontal cells processes into the outer nuclear layer in *Bbs8*^*floxed/floxed*^*; Rho-Cre*^+^ mice (Fig. 14). However, the horizontal cell phenotype in 6 months old *Bbs8*^*floxed/floxed*^*; Rho-Cre*^+^ mice appears mild even though the state of degeneration in these animals is much more advanced than in congenital BBS mutant mice at P21. Therefore, even though retinal degeneration does trigger intrusion of horizontal cell processes into the outer nuclear layer over time, the severity of the phenotype observed in young congenital BBS mutant mice is not recapitulated in *Bbs8*^*floxed/floxed*^*; Rho-Cre*^+^ mice at any age, even when compared to *Bbs8*^*floxed/floxed*^*; Rho-Cre*^+^ mice at a much more advanced stage of retinal degeneration.

The phenotypic severity of the horizontal cell phenotype observed in young congenital BBS mutant mice is not fully recapitulated by the inactivation of BBSome function in photoreceptors alone. Therefore, the horizontal cell phenotype is not solely a consequence of retinal degeneration. Instead, these results indicate that the aberrant intrusion of horizontal cell processes is due to the loss of a novel cell-autonomous role of the BBSome in horizontal cells, in addition to the effects of retinal degeneration.

## Discussion

The absence of BBSome function significantly impairs the formation of photoreceptor outer segments during photoreceptor terminal differentiation^20^. In addition, having a dysfunctional BBSome causes proteins normally excluded from the photoreceptor outer segment to inundate the outer segment^22^. Since the photoreceptor outer segment is a modified cilium, it is not surprising that the BBSome is integral for protein trafficking in the outer segment. However, whether the absence of BBSome function affects synaptogenesis was an unexplored question. In this study, we found that the lack of BBSome function both decreases ribbon synaptic density and alters synaptic positioning in the retina, causing ectopically localized ribbon synapses within the outer nuclear layer. We found that both bipolar and horizontal cells aberrantly extend processes into the outer nuclear layer in BBS mutant retinas at very early stages of degeneration. The aberrant intrusion of horizontal cell processes is especially severe, reaching far beyond the aberrant bipolar cell processes. In 21-day old BBS mutant retinas where degeneration is still mild, some horizontal cell processes are observed spanning the entire thickness of the outer nuclear layer, and these intruding processes are decorated with ectopic ribbon synapses located deep within the outer nuclear layer.

One explanation for the intrusion of aberrant horizontal cell processes is that it is a consequence of retinal degeneration. Examples of ectopic synapses can be found in mouse models with mutated components of the ribbon synapse. In *Nob2* mutant mice in which one of the subunit of the L-type calcium channel is non-functional, ectopic ribbon synapses are observed in the outer nuclear layer and are proposed to result from the retraction of photoreceptor axons^39^. In these mice, retinal bipolar cells and not horizontal cells constitute the main component of these ectopic synaptic contacts, although both cells types extend aberrant processes into the outer nuclear layer. In Bassoon mutant mice, numerous ectopic synapses form within the outer nuclear layer due to the aberrant sprouting of their horizontal and bipolar cells processes, seen as a part of the remodeling process^40^. In this mouse model, the ectopically localized synapses found closer to the outer plexiform layer are accounted for by axon retraction of the photoreceptor cells, but those found distant from the outer plexiform layer are thought to be the consequence of *de novo* formation of ectopic synapses as many of these distant synapses lack contact with bipolar cell processes^40^. Notably, in this study, aberrant intrusion of horizontal cell processes is not observed in *Bbs8*^*floxed/floxed*^*; Rho-Cre*^+^ mice up to 3 months of age despite retinal degeneration in these mice. Even at up to 6 months, when greater than 50% of photoreceptor cells are lost in *Bbs8*^*floxed/floxed*^*; Rho-Cre*^+^ mice, the intrusion of horizontal cell processes induced by retinal degeneration is relatively mild compared to the phenotype observed in young congenital BBS mutant mice. These observations point to the possibility that the BBSome plays a cell-autonomous role in horizontal cells, and that the phenotype is not solely caused by retinal degeneration. In congenital BBS mutant mice, two overlapping phenomena likely contribute to the intrusion of horizontal cell processes into the outer nuclear layer. First, during retinal synaptogenesis, the axonal pathfinding of horizontal cells is negatively impacted due to the lack of BBSome function. Second, photoreceptor loss over time exacerbates the intrusion of aberrant horizontal cell processes as a part of the retinal remodeling process. In retinas with photoreceptor-specific deletion of BBS8 by Rho-*Cre*, only the second phenomenon occurs, but not the first. Therefore, the phenotype is only observed in *Bbs8*^*floxed/floxed*^*; Rho-Cre*^+^ mice with advanced stages of retinal degeneration.

It is worth noting that retinal degeneration in *Bbs8*^*floxed/floxed*^*; Rho-Cre*^+^ mice is slower than in *Bbs8*^−/−^ congenital knockout mice. There are several explanations underlying the different rates of retinal degeneration in congenital *Bbs8*^−/−^ mice and *Bbs8*^*floxed/floxed*^*; Rho-Cre*^+^ mice. First, in a reporter line consisting of a β-galactosidase gene controlled by a floxed transcription stop signal, the deletion of the floxed site is not complete until about P18 in this rhodopsin-Cre mouse line^30^, whereas BBS8 is congenitally absent in *Bbs8*^−/−^ retinas. Therefore, the initial presence of BBS8 in photoreceptors during the second-third postnatal week may allow the development of normal photoreceptor outer segments and delay the onset of retinal degeneration. Second, cones are not affected by genetic recombination induced by rhodopsin promoter driven CRE in this mouse line^30^. Therefore, the delay in cone degeneration in *Bbs8*^*floxed/floxed*^*; Rho-Cre*^+^ mice may have caused their slower overall retinal degeneration. Lastly, the BBSome may be required in cell types other than photoreceptor cells in the retina, and therefore the inactivation of the BBSome in photoreceptor cells alone may not fully recapitulate the severity of retinal degeneration caused by the global loss of BBSome function in congenital knockout mouse lines. Investigating whether the BBSome is required in cell types other than photoreceptors has important implications for the design of gene therapies targeting blindness in BBS.

Thus far, retinal degeneration in BBS is attributed to the impaired survival of the photoreceptors, whereas the inner retina is considered to be relatively intact even as degeneration progresses. As a result, gene therapy efforts in BBS are concentrated on rescuing photoreceptor cells alone, frequently employing gene vectors containing photoreceptor-specific promoters such as rhodopsin to drive exogenous gene expression. This report offers evidence of potential roles of the BBSome in other cell types in the retina. Whether the lack of BBSome function in non-photoreceptor cells in the retina merely causes a cellular dysfunction without contributing to photoreceptor degeneration or the dysfunction exacerbates photoreceptor degeneration is an important question to the optimal design of gene therapies.

On the other hand, it is unclear how loss of BBSome function impairs ribbon synapse formation in photoreceptor cells. Recent evidence suggests that BBS proteins are present at neuronal synapses in the brain^19^. Loss of BBS4, a member of the BBSome complex, causes reduced dendritic lengths as well as reduced dendritic spine count in neurons^19^. Based on these pieces of evidence, a potential role of the BBSome complex in trafficking receptors in dendritic spines has been proposed. The BBSome is also required for axonal targeting in olfactory neurons^18^. Therefore, the BBSome could potentially play a direct role at the photoreceptor synapse. However, the decreased retinal synaptogenesis in BBS mutant mice could also be a consequence of having reduced quantities of proteins important for synaptogenesis due to the massive protein mislocalization to the photoreceptor outer segment that is known to occur^22^. Alternatively, a global alteration in transcription caused by altered ciliary signaling in photoreceptors could also indirectly impact synaptogenesis. Therefore, whether the BBSome is directly involved in functions at the photoreceptor synapse remains unclear and merits further investigation.

Lastly, how the guidance of axonal processes of horizontal cells is affected in the absence of BBSome function is unknown. Beyond ciliary signaling, a growing body of work has demonstrated the role of the BBSome in regulating receptor localization and signaling on the neuronal plasma membrane in a cilia-independent manner^15^. These works contribute to an emerging picture containing both classical ciliary functions and novel, non-ciliary BBSome functions in neurons. Wandering axons have been observed in the olfactory bulb of mice lacking BBS8, a member of the BBSome^18^, as well as in olfactory sensory neurons lacking IFT88, a member of the IFT complex B, which plays a role in anterograde trafficking^41^. In this report, we propose that the intruding, or wandering horizontal cell axons in the outer nuclear layer could be due to cell-autonomous roles of the BBSome in these inner neurons of the retina. The connection between neuronal axons and their intended targets requires a series of steps involving signaling and pathfinding using receptors on the membrane, and many of these receptors are still unknown. It is possible that the BBSome is required for trafficking certain receptors to neuronal axons important for sensory input in pathfinding, and the lack of such receptors may cause their axons to be mistargeted, leading to aberrant synaptic positioning. This work adds to the possibility that the BBSome can be required, in certain cases, for interpreting attractive or inhibitory signals for neurons to establish synaptic contacts with their intended target in order to achieve proper synaptic positioning. This work paves the way for understanding the novel role of the BBSome in synaptogenesis and axonal targeting in neurons.

## Data Availability

All data generated or analyzed during this study are included in this manuscript.
